# Chronic wasting associated with *Chlamydia pneumoniae* in three ex situ breeding facilities for tropical frogs

**DOI:** 10.1007/s10482-020-01483-6

**Published:** 2020-11-05

**Authors:** Tobias Eisenberg, Ahmad Fawzy, Ute Kaim, Anne Nesseler, Karin Riße, Iris Völker, Silke Hechinger, Nicole Schauerte, Christina Geiger, Tobias Knauf-Witzens, Ingo Schwabe, Christiane Schnee, Elisabeth Liebler-Tenorio, Can Imirzalioglu, Reinhard Sting

**Affiliations:** 1 Department of Veterinary Medicine, Hessian State Laboratory (LHL), Schubertstr. 60, 35392 Giessen, Germany; 2 Faculty of Veterinary Medicine, Department of Medicine and Infectious Diseases, Cairo University, Cairo, Egypt; 3 Frankfurt Zoo, Bernhard-Grzimek-Allee 1, 60316 Frankfurt, Germany; 4 Wilhelma – The Zoological and Botanical Gardens, Wilhelma 13, 70376 Stuttgart, Germany; 5 Chemical and Veterinary Analysis Agency Stuttgart, Schaflandstr. 3/3, 70736 Fellbach, Germany; 6 Institute of Molecular Pathogenesis (IMP), Friedrich-Loeffler-Institut (FLI), Federal Research Institute for Animal Health, Naumburger Str. 96 a, 07743 Jena, Germany; 7 Justus Liebig University Giessen and German Center for Infection research (DZIF), Partner-site Giessen-Marburg-Langen, Institute for Medical Microbiology, Schubertstr. 81, 35392 Giessen, Germany; 8 Institute of Hygiene and Infectious Diseases of Animals, Justus-Liebig-University Giessen, 35392 Giessen, Germany

**Keywords:** *Chlamydia pneumoniae*, Frog, Amphibian, Infectious disease, Zoonosis

## Abstract

A number of different *Chlamydia* spp. have been detected in the class Amphibia with *C. pneumoniae* being the predominant species involved. Chlamydiae have been linked to mass mortality events, thereby representing significant pathogens that deserve attention with respect to worldwide amphibian decline. We here present six cases of chlamydiosis and asymptomatic chlamydial infections in different frog species from three ex situ amphibian conservation facilities. Clinical signs predominantly characterised by regurgitation, chronic wasting, lethargy and suspended breeding were associated with *C. pneumoniae* infection. Despite various treatment regimens, it was not possible to clear infections. However, *intra vitam* diagnostics succeeded from skin, faeces and urine for the first time.

## Introduction

The genus *Chlamydia* is the sole genus in the family *Chlamydiaceae* and currently contains thirteen valid taxa (Borel and Greub 2019; Kuo et al. 2011; Sachse et al. 2015), although a novel genus, *Chlamydophila,* had transitionally been accepted between 1999 and 2015 (Everett et al. 1999). Besides traditional avian and mammalian hosts, chlamydiae are known to infect also poikilothermic vertebrates including fish, amphibians and reptiles (Borel et al. 2018; Homer et al. 1994; Huchzermeyer et al. 1994; Schindarow and Tonew 1965). Molecular techniques have facilitated detection of chlamydiae in various tissues. In contrast to clinical cases (chlamydiosis) such proofs especially query the significance and a supposed pathogenic potential in asymptomatic chlamydial infections. Recently, a number of candidate species have been proposed, but their formal description as valid species is still pending (Taylor-Brown et al. 2017).

Chlamydiosis in amphibians associated with a mass mortality event has first been described in a commercial breeding facility for African clawed frogs in the early 1980s (Newcomer et al. 1982). A *Chlamydia* sp. was identified as the causative agent of this mortality based on phenotypic methods including electron microscopy, immunofluorescence and cell culture (Newcomer et al. 1982; Wilcke et al. 1983). Although gross pathological lesions were largely missing, a representative strain from this outbreak turned out to be lethal for chicken embryos and naive frogs, but did not kill mice upon peritoneal inoculation (Wilcke et al. 1983). Pathological changes observed in a similar case also in African clawed frogs have been documented (Howerth 1984) and included hepatomegaly, distention of the gall bladder, splenomegaly, degenerative and inflammatory changes in the kidneys, epicarditis and myocarditis. In this mortality event, histopathology revealed mononuclear cell infiltrates and necrosis of hepatocytes in each necropsied frog, accompanied by occasional other changes of hepatocytes, reticuloendothelial cell proliferation, lymphoid depletion and spleen necrosis (Howerth 1984). However, types of inflammation varied greatly in histopathology between studies, among others including acute (Howerth 1984), lymphohistiocytic (Blumer et al. 2007), mononuclear (Newcomer et al. 1982) and lymphocytic to granulomatous (Reed et al. 2000) states. A complete overview of cases of amphibian chlamydioses from the literature is depicted in Table 1. Later, *C. pneumoniae* has been identified as causative agent of disease in amphibians (Berger et al. 1999; Bodetti et al. 2002; Fratzke et al. 2019; Reed et al. 2000) and confirmed by molecular methods (Berger et al. 1999). An Australian giant barred frog (*Mixophyes iteratus*) suffering from a severe, chronic, mononuclear pneumonia, non-regenerative anaemia and pancytopenia represents the first chlamydiosis case from the wild. This frog was found to be infected with a ‘homeothermic‘*C. pneumoniae* strain giving rise to the question whether amphibians may play a role for zoonotic infections (Berger et al. 1999; Wright 1996). A further outbreak in African clawed frogs with significant morbidity and mortality due to *C. pneumoniae* was noted, but the most prominent clinical signs—lethargy, bloating and sloughing skin—might have been exacerbated by stress during importation and chytridiomycete co-infection (Reed et al. 2000). The majority of studies describes chlamydiae in anuran hosts (frogs), but urodelians (salamanders) were occasionally found to harbour not yet classified chlamydial species (Martel et al. 2012). Although all salamanders from three different species suffering from anorexia, lethargy, oedema and markedly abnormal gait died without any gross pathological lesions, a putatively novel member of the *Chlamydiaceae* most closely related to unclassified chlamydial species from fish and bullfrogs could be detected in liver tissue by 16S rRNA gene sequencing (Martel et al. 2012, 2013). Lastly, a retrospective case study from Switzerland had found other chlamydial species besides *C. pneumoniae* that were closely related to *C. suis* and *C. abortus* in a number of paraffin embedded tissue samples from native as well as exotic amphibians (Blumer et al. 2007). Another case series describes an exclusively neurotropic form of fatal *C. pneumoniae* infections in a breeding colony of endangered Houston toads (*Anaxyrus houstonensis*) in a zoological collection (Fratzke et al. 2019). Clinical signs included ataxia, anisocoria, and decreased righting reflex. Necrotising and histiocytic polioencephalomyelitis and ganglionitis were the major pathological findings and chlamydial inclusions were found within and in the surrounding of neurons (Fratzke et al. 2019). In addition, infections with members of the *Parachlamydiaceae* (genera *Parachlamydia* Bates et al. 2018; Vajana et al. 2019 and *Neochlamydia* Vajana et al. 2019) and *Simkaniaceae* (Vajana et al. 2019) have occasionally been reported.

**Table 1:** Case series presentation of chlamydial infections in amphibians from the literature

Case no.	Amphibian species	No. of animals involved	Country (infection noticed in the wild [w] or in captivity [c])	Tissues with chlamydial proof	Chlamydial species	Year	Source
01	African clawed frog (*Xenopus laevis*)	20.000	USA (c)	Liver, spleen, kidney, lung, heart	“*C. psittaci*“	1982	Newcomer et al. (1982), Wilcke et al. (1983)
02	African clawed frog (*Xenopus laevis*)	6	USA (c)	Liver, spleen, kidney, heart	“*C. psittaci*“	1984	Howerth (1984)
03	Eyelash leaf frogs (*Cornufer* [*Ceratobatrachus*] *guentheri*)	8	Canada, imported from Salomon Islands (w)	Liver, spleen, kidney, lung, heart, muscle, fat tissue, medulla	*Chlamydia* sp.	1992	Honeyman et al. (1992)
04	*Xenopus laevis, „*Bufo maculatum*“, Pachytriton* sp.	15	Germany, (c)	Liver, spleen, kidney, lung, heart, blood cells	“*C. psittaci*“	1998	Mutschmann (1998)
05	Giant barred frog (*Mixophyes iteratus*)	1	Australia, (w)	Lung, blood	*C. pneumoniae*	1999	Berger et al. (1999)
06	African clawed frog (*Xenopus tropicalis*)	220	USA, imported from West Africa (c)	Liver	*C. pneumoniae*	2000	Reed et al. (2000)
07	Blue Mountains tree frogs (*Litoria citropa*)	2	Australia (c)	Kidney, heart, lung, gastrointestinal tract, brain	*C. pneumoniae*	2002	Bodetti et al. (2002)
08	*Cryptohylax gresshoffi*	3	Central African Republic (w; [Germany])	Liver, spleen, kidney	*C. pneumoniae*	2001	Hotzel et al. (2001)
09	*Rana temporaria* (mass mortality 1991/1992)	8 out of 54 samples (no. of individuals unknown)	Switzerland (w)	Kidney, spleen, liver	*“C. suis“* (98–99% homology)	2007	Blumer et al. (2007)
10	Various species (sudden deaths in zoos/private collections	3 out of 83 samples (no. of individuals unknown)	Switzerland (c)	Kidney, spleen, liver	*C. pneumoniae*	2007	Blumer et al. (2007)
11	*Xenopus laevis* (healthy control)	3 out of 38 samples (n = 19 individuals)	Switzerland (c)	Kidney, spleen, liver	2 × *“C. suis“* (98–99% homology), 1 × endosymbiont of *Acanthamoeba* sp.	2007	Blumer et al. (2007)
12	*Rana temporaria* (2004/2005)	6 out of 238 samples (n = 126 individuals)	Switzerland (w)	Kidney, spleen, liver	*“C. suis“* (98–99% homology), 1x *C. pneumoniae,* 3 × *C. abortus*, 1 × uncultured Chlamydiales	2007	Blumer et al. (2007)
13	*Salamandra corsica* (Corsican fire salamander)	5	The Netherlands/Germany (c)	Liver	‘*Candidatus* Amphibiichlamydia salamandrae’	2012	Martel et al. (2012)
14	*Neurergus crocatus* (yellow spotted newt)	11 (from 3 collections)	The Netherlands/Germany (c)	Liver	‘*Candidatus* Amphibiichlamydia salamandrae’	2012	Martel et al. (2012)
15	*Neurergus strauchii* (Strauch’s spotted newt)	6 (from 2 collections)	The Netherlands/Germany (c)	Liver	‘*Candidatus* Amphibiichlamydia salamandrae’	2012	Martel et al. (2012)
16	*Lithobates catesbeianus* (North American bullfrog tadpoles)	No clinical signs (71% prevalence)	The Netherlands (w; introduced population)	Liver	‘*Candidatus* Amphibiichlamydia ranarum’	2013	Martel et al. (2013)
17	*Anaxyrus houstonensis* (Houston toad)	175	USA (c)	Brain, (liver)	*C. pneumoniae*	2019	Fratzke et al. (2019)

In the present study, we have compiled clinical data on chlamydial infections in amphibians from the literature with those from own actual cases. The data of the present study was obtained from frogs mostly developing clinically signs, however, this was not a designed study. Therefore, the available data vary from case to case and could not be consistently collected. Even so, cluster of cases in tropical frog holdings attracting attention by clinical, pathological, histological and microbiological findings and the presence of chlamydiae in organ samples advocate that these pathogens have the potential to impair the health of amphibians. The scope of the present study was to report the detection of chlamydiae in tropical frogs suffering from debilitating disease. We argue that rather non-specific behavioural abnormities have to be taken into consideration that finally led to the loss of valuable breeding groups of endangered amphibian species.

## Materials and methods

### Collections and animals

Observations described in this manuscript were made on animals from three different holdings, namely one private collection and two zoos, designated as zoo A and zoo B, respectively. Each of the groups of frogs was housed in a terrarium (60 × 65 × 120 cm; LxWxH), densely planted with tropical vegetation. Maintenance conditions included a twelve-hour night/day interval with temperatures between 20 and 27 °C during the day unless otherwise stated. An automatic sprinkling system was used to achieve a relative humidity above 70%.

The private collection comprised five captive-bred approx. 4-year old coronated tree frogs, *Triprion* (*Anotheca*) *spinosus,* originally imported from Costa Rica housed together with eight lemur leaf frogs (*Agalychnis lemur*). The *Triprion* group was later transferred to the quarantine unit of zoo B. Additionally, there was a second terrarium with a group of eight captive-bred, 3-year old fringed leaf frogs (*Cruziohyla craspedopus*), originally imported from Peru. A third terrarium contained a group of 17 captive-bred 1–3-year old casque-headed treefrogs (*Triprion petasatus*). All three terraria were standing directly next to each other, but drains were closed off each by a turncock to prevent wastewater bypass. The *Triprion petasatus* terrarium was further equipped with an additional ultraviolet and heating device (Bright Sun, 50 W, Lucky Reptile, Germany) to achieve daytime maximum temperatures of up to 35 °C. In zoos A and B, each a group of White’s tree frogs, *Ranoidea* (*Litoria*) *caerulea,* and long-nosed horned frogs (*Megophrys nasuta*), 2 females and one male, were kept without any clinical abnormalities under the same conditions like in the private collection. Zoo B also took over the group of *Triprion spinosus* from the private collection.

All the mentioned frog species of the three collections were initially checked during quarantine and when abnormalities were observed by specific qPCRs for the presence of *Batrachochytrium dendrobatidis, B. salamandrivorans*, ranavirus, *Brucella* spp. and by faecal examination for parasites and yielded—except for the *Strongyloides* sp.—only negative results (data not shown).

### Necropsy and histopathology

A full necropsy was performed on deceased frogs from this study. For histopathological examination, small slices of multiple organs were fixed in buffered 4% formalin, processed by standard methods and embedded in paraffin. Sections were stained with hematoxylin–eosin (HE). Additional sections were further processed for *Chlamydia*-specific immuno-histochemistry (IHC). Briefly, chlamydiae were detected by the indirect immunoperoxidase method. Tissues examined included skin, muscle, bone, eye, brain, heart, lung, liver, spleen, kidney, intestine, mesothelium, testis and ovary. For antigen retrieval, slides were incubated in 0.025% proteinase K (Sigma, Taufkirchen, Germany) in phosphate buffered saline (pH 7.4) for 5 min at 37 °C. A monoclonal antibody against *Chlamydia*-LPS (ACIP500, Progen Biotech GmbH, Heidelberg, Germany) was used as primary antibody, HRP-conjugated goat anti-mouse immunoglobulins (Agilent Dako Products, Waldbronn, Germany) as secondary antibody and 3-amino-9-ethlycarbazole as chromogen. Sections were counterstained with hemalaun.

### Chlamydial culture and bacterial cultivation from clinical samples

For chlamydia isolation, buffalo green monkey (BGM) cells in Eagle’s Minimal Essential Medium (EMEM, Lonza, Basel, Switzerland) with 2 mmol glutamine and 5% fetal calf serum were seeded into Trac bottles with coverslips (Sterilin, Thermo Fisher, Dreieich, Germany) and incubated at 37 °C with 5% CO_2_ for 3–4 days. Tissue samples from liver, spleen and kidney were homogenised and ultrasonicated in 2–5 mL SPGA (sucrose-phosphate-glutamine-albumine) stabilising buffer. After a 15 min centrifugation at 500 × *g*, 30–300 µL of the supernatants were inoculated into 4–6 Track bottles and centrifuged at 3400 × *g* at 37 °C for 60 min. The bottles were incubated for 2 h and EMEM was replaced by serum-free UMDCK medium (Lonza) with an antibiotic cocktail containing nystatin, gentamicin and vancomycin. Medium was renewed after 18 h. Two to 3 days after inoculation, the coverslips were fixed with methanol and cells were stained with fluorescence-labeled monoclonal antibodies from the IMAGEN *Chlamydia* kit (Oxoid).

Samples from living frogs (skin swabs, faeces, urine, cloacal washings) or various tissue samples (liver, spleen, kidney, lung, ovary, intestine, skin and in one case intraocular fluid) obtained during necropsies were directly cultured on 5% Columbia sheep blood (SBA) and water-blue metachrome-yellow lactose agar according to Gassner (all Oxoid, Wesel, Germany) at 20 °C aerobically for up to 48 h. Concomitant bacterial isolates were identified by MALDI-TOF mass spectrometry (MALDI-TOF MS, Bruker Biotyper, Bruker Daltonik, Bremen, Germany). Tissue samples were also inoculated onto *Brucella* selective media (with and without crystal violet [Oxoid]) as well as in enrichment broth for the isolation of *Salmonella*.

### *Chlamydia* detection by PCR and phylogenetic analysis

For the molecular detection of chlamydiae, 250 mg tissue samples as mentioned above for bacterial cultivation were homogenised in 1 mL of phosphate buffered saline (PBS, Oxoid) using a FastPrep-24 Classic bead beating grinder and Lysing Matrix tubes with ceramic beads (MP Biomedicals, Eschwege, Germany). Homogenates were centrifuged briefly to sediment tissue fragments and DNA was extracted from 200 µL supernatant via IndiMag Pathogen Kit on the IndiMag 48 Magnetic bead based nucleic acid extractor (Indical, Leipzig, Germany) according to the manufacturer’s instructions.

Detection of chlamydial DNA was carried out by qPCR according to (Anonym 2020) based on the 23S rRNA gene (genus level), whereas *Chlamydia* typing was performed with a commercial qPCR assay (Allplex RP-4, Seegene, Germany) based on the *murB* gene (species level *C. pneumoniae*). An almost full-length 16S rRNA gene (> 1500 bp) was amplified using the primers 16S F (5′GCGTGGATGAGGCATGCAA′3) and 16S R (5′GGAGGTGATCCAGCCCCA′3) described by Everett (2000). The PCR reaction (25 µL) consisted of 12.5 µL 2X Platinum™ SuperFi™ PCR Master Mix (Thermo Fisher Scientific, Germany), 1.25 µL 16S F primer (10 µM), 1.25 µL 16S R primer (10 µM), 5 µL PCR water and 5 µL DNA. Cycling conditions were as following: 1 × (98 °C–30 s) 45 × (98 °C–10 s; 65 °C–10 s; 72 °C–90 s) 1 × (72 °C–5 min). PCR products were sent to Seqlab-Microsynth laboratories (Göttingen, Germany) for DNA purification and sequencing.

Using the sequence obtained in this study together with homologues of other *Chlamydia* species, we carried out a phylogenetic analysis with PhyML (Maximum-Likelihood algorithm, 100 bootstraps, http://www.phylogeny.fr/one_task.cgi?task_type=phyml) under default conditions (Dereeper et al. 2008). For an overview of the different diagnostic approaches employed for chlamydial identification at genus or species level in this as well as in comparable earlier studies the reader is referred to Table 2.

**Table 2:** Diagnostic methods used for the detection of chlamydial infections in amphibians from the literature and from this study

Case no.	Amphibian species	Histo^1^	IHC^2^	IF^3^	TEM^4^	LPS^5^	CC^6^	ECE^7^	MIT^8^	16S^9^
01	*Xenopus laevis*	X		x	x		x	x	x	
02	*Xenopus laevis*	x			x					
03	*Cornufer* (*Ceratobatrachus*) *guentheri*	x			x					
04	*Xenopus laevis, „*Bufo maculatum*“, Pachytriton* sp.	x		x						
05	*Mixophyes iteratus*	x		x	x	x	x			x
06	*Xenopus tropicalis*	x		x	x		x			x
07	*Litoria citropa*	x	x	x						x
08	*Cryptohylax gresshoffi*	x		x			x			x
09	*Rana temporaria*	x	x							x
10	Various species	x	x							x
11	*Xenopus laevis*	x	x							x
12	*Rana temporaria*	x	x							x
13	*Salamandra corsica*		x		x					x
14	*Neurergus crocatus*		x		x					x
15	*Neurergus strauchii*		x		x					x
16	*Lithobates catesbeianus*			x						x
17	*Anaxyrus houstonensis*	x			x		x			
18	*Ranoidea* (*Litoria*) *caerulea*	x	x							
19	*Triprion* (*Anotheca*) *spinosus*	x	x				x			x
20	*Agalychnis lemur*	x								
21	*Cruziohyla craspedopus*	x								
22	*Triprion petasatus*	x					x			
23	*Megophrys nasuta*	x								

Case no.	16-23S^10^	*ompA*^11^	*ompB*^12^	*groESL*^13^	ChlPCR^14^	CpPCR^15^	CsPCR^16^	LAS^17^	rtfPCR^18^	Source
01										Newcomer et al. (1982), Wilcke et al. (1983)
02										Howerth (1984)
03										Honeyman et al. (1992)
04										Mutschmann (1998)
05		x	x							Berger et al. (1999)
06		x								Reed et al. (2000)
07		x								Bodetti et al. (2002)
08	x	x	x	x						Hotzel et al. (2001)
09		x								Blumer et al. (2007)
10		x								Blumer et al. (2007)
11		x								Blumer et al. (2007)
12		x								Blumer et al. (2007)
13										Martel et al. (2012)
14										Martel et al. (2012)
15										Martel et al. (2012)
16								x		Martel et al. (2013)
17									x	Fratzke et al. (2019)
18					x	x				This study
19					x	x				This study
20					x					This study
21					x					This study
22					x					This study
23					x					This study

^1^Histology; ^2^ immuno-histochemistry; ^3^ direct immunofluorescence; ^4^ transmission electron-microscopy; ^5^ LPS antigen detection assay; ^6^ cell culture; ^7^ embryonated chicken eggs; ^8^ mouse inoculation test; ^9^ 16S rRNA gene; ^10^ 16-23S intergenic spacer genes; ^11^ *omp*A gene sequencing; ^12^ *omp*B gene sequencing; ^13^ *groESL* gene sequencing; ^14^ *Chlamydia* spp. qPCR; ^15^ *C. pneumoniae* PCR; ^16^ *C. suis* PCR; ^17^ laser capture microdissection; ^18^ real-time fluorescence resonance energy transfer PCR

### Further testing

Skin and liver or kidney samples were tested for the presence of chytrid fungi (*Batrachochytrium* [*B*.] *dendrobatidis, B. salamandrivorans*) and ranavirus by PCR, respectively, employing previously published protocols (Black et al. 2017; Boyle et al. 2004).

## Results

### Case history, clinical and pathological observations

1. Private collection:

*Triprion* (*Anotheca*) *spinosus* and *Agalychnis lemur*: The group of coronated treefrogs regularly laid fertilised clutches until summer 2018. Whereas the lemur leaf frogs did not show any clinical signs or abnormalities, the *Triprion* group stopped calling and breeding, lost their bright skin colouration and developed a faded and dry skin with regular ecdysis and consistently regurgitated insect prey items within 48 h of feeding. At this stage, the group was transferred to the quarantine unit of zoo B in February 2019 in order to improve breeding success.

*Cruziohyla craspedopus*: The group regularly laid fertilised clutches until summer 2018. Similarly, frogs stopped calling and breeding with no externally visible signs like weight loss and skin abnormalities, except sporadically regurgitating insect prey items. All animals survived but without any breeding success.

*Triprion petasatus*: As these frogs are very seasonal, calling cannot be observed throughout the year and cannot be exploited for assessment of clinical disease status. Breeding could be induced every year. The group was active, but often regurgitated insect prey items since summer 2018 and emaciated significantly. However, the group also contained subjects with an adipose nutritional status. All these frogs were still active at night. Ten frogs died, but only two were available for post mortem examinations due to high-grade decomposition; the rest of the animals survived. A low-grade nematode infestation with *Strongyloides* sp. was noted which turned out to be refractory to oral levamisole and ivermectin treatment regimens (data not shown).

2. Zoo A:

*Ranoidea* (*Litoria*) *caerulea*: During 2016 and 2017, 13 White’s tree frogs died. Prior to death, three animals displayed clinical signs including emaciation, oedema on legs and abdomen, neurological signs, and bloating (Table 3).

**Table 3:** Summarised results of involved frog species from this study, kept in German ex situ breeding programs, and their clinical and necropsy findings and chlamydial test results

Case no.	Amphibian species	Breeding location	No. of submitted/no. of involved frogs	Clinical signs	Gross pathology	Laboratory ID (sample matrix)	*Chlamydia* spp. qPCR^2^ result (sample matrix [Ct value])	*Chlamydia pneumoniae* qPCR result (Ct value)	IHC^1^	Cell culture	Proof of chlamydial species (for the case)
18	*Ranoidea* (*Litoria*) *caerulea*	Zoo A	3/13	Emaciated (n = 2); oedematous legs and abdomen (n = 2); neurologic signs (n = 1); bloating (n = 1)	erythema of the ventral skin (n = 1); swollen liver (n = 2); *Strongyloides* sp. (moderate; n = 1); acute multifocal suppurative nephritis (n = 1)	A17077031 A17034507 161015325 171000715 A17020008 A19125593	+ (pooled tissues [34.2]) + (faecal sample [24.5]) + (faecal sample [32.6]) + (faecal sample [38.9]) + (skin samples [35.0]) ? (skin sample [40.5])	+ (28.0) n.d. n.d. n.d. − n.d.	− n.d. n.d. n.d. − n.d.	n.d. n.d. n.d. n.d. n.d. n.d.	*C. pneumoniae*
19	*Triprion* (*Anotheca*) *spinosus*	Private collection, zoo B	4/5	CW, I	Cachexy (n = 5)	191006340 191006501 191008608 191010174 191007396 191007397 191007396 191007397 191007396 191007397	+ (pooled tissues [29.0]) + (pooled tissues [31.0]) + (pooled tissues [30.0]) + (pooled tissues [34.8]) ? (oral sample [41.1]) − (oral sample) ? (skin swab [41.1]) − (skin swab) − (cloacal washing) − (cloacal washing)	− + (29.2) n.d. n.d. n.d. n.d. n.d. n.d. n.d. n.d.	− + + + n.d. n.d. n.d. n.d. n.d. n.d.	− − n.d. n.d. n.d. n.d. n.d. n.d. n.d. n.d.	*C. pneumoniae*
20	*Agalychnis lemur*	Private collection	0/15	None	n.d.	191008622	+ (urine sample [35.6])	n.d.	n.d.	n.d.	*Chlamydia* sp.
21	*Cruziohyla craspedopus*	Private collection	0/8	I	n.d.	191008622	+ (urine sample [37.5])	n.d.	n.d.	n.d.	*Chlamydia* sp.
22	*Triprion petasatus*	Private collection	2/17	CW	Cachexy and *Strongyloides* sp. (low-grade; n = 2)	191017444 191015724 191008622	+ (pooled tissues [30.0]) + (pooled tissues [35.6]) + (urine sample [35.0])	n.d. n.d. n.d.	n.d. n.d. n.d.	− n.d. n.d.	*Chlamydia* sp.
23	*Megophrys nasuta*	Zoo B	2/3	Sudden death	Cachexy and pyogranulomatous tissue inflammation (n = 1); ovar haemorrhage and follicle necrosis (n = 1)	171006016 191013315	+ (pooled tissues [33.8]) + (pooled tissues [31.4])	n.d. −	n.d. n.d.	n.d. n.d.	*Chlamydia* sp.

^1^IHC, immuno-histochemistry; ^2^qPCR according to Anonym (2020); +, positive; ?, questionable; −, negative; n.d., not determined; CW, chronic wasting; I, infertility

3. Zoo B:

*Triprion spinosus*: During quarantine between March and May 2019, three of the frogs originating from the private collection died. The two remaining frogs displayed wasting, emaciated nutritional status. Despite antimicrobial therapy as described below they finally died nonetheless two to 3 weeks later.

*Megophrys nasuta*: Two adult females were suddenly found dead one at a time on different days. They did not show any conspicuous clinical signs prior to death as was the case for the remaining male individual from the same breeding group.

Available background information on the cases from this study as well as previous, comparable infection events are summarised in Tables 1, 2, 3 and 4.

**Table 4:** Organ distribution of *Chlamydia* detected by immuno-histochemistry

Case no.	Species	Laboratory ID	Skin	Muscle	Bone	Eye	Brain	Heart	Lung	Liver	Spleen	Kidney	Intestine	Mesothelium	Testis	Ovary
18	*Litorea caerulea*	A17020008	–	–	n.d.	n.d.	n.d.	n.d.	n.d.	–	–	n.d.	–	–	n.d.	n.d.
18	*Litorea caerulea*	A17077031	–	–	n.d.	n.d.	–	–	–	n.d.	–	–	n.d.	n.d.	n.d.	–
19	*Triprion spinosus*	191006340	–	–	–	n.d.	n.d.	n.d.	n.d.	–	n.d.	n.d.	–	–	n.d.	n.d.
19	*Triprion spinosus*	191006501	+	–	–	–	n.d.	n.d.	n.d.	–	n.d.	+	+	–	–	n.d.
19	*Triprion spinosus*	191008608	–	–	–	n.d.	n.d.	n.d.	n.d.	–	n.d.	+	–	–	n.d.	n.d.
19	*Triprion spinosus*	191010174	–	–	–	n.d.	n.d.	n.d.	+	–	n.d.	–	–	–	–	n.d.

+, positive; −, negative; n.d., not determined

### Treatment

In zoo B, parenteral treatments were initiated in the coronated tree frogs during first *Chlamydia* proofs within the quarantine period. For this purpose, injections with enrofloxacin (Baytril 2.5%, Bayer, Leverkusen, Germany) into the dorsal lymph sac at 5 mg/kg SC q 24 h were conducted. In the private collection, treatment baths were initially (in March 2019) carried out to treat *Agalychnis lemur*, *Cruziohyla craspedopus* and *Triprion petasatus* with tetracycline (Ursocyclin, Serumwerk Bernburg, Bernburg, Germany; 100 mg/L for 60 min q 24 h for 12 days) and subsequently—due to repeated pathogen detection in April 2019—with enrofloxacin (Enro-Sleecol, Dechra, Aulendorf, Germany; 500 mg/L for 6 h q 24 h for 12 days).

### Immuno-histochemistry

**Fig. 1:**
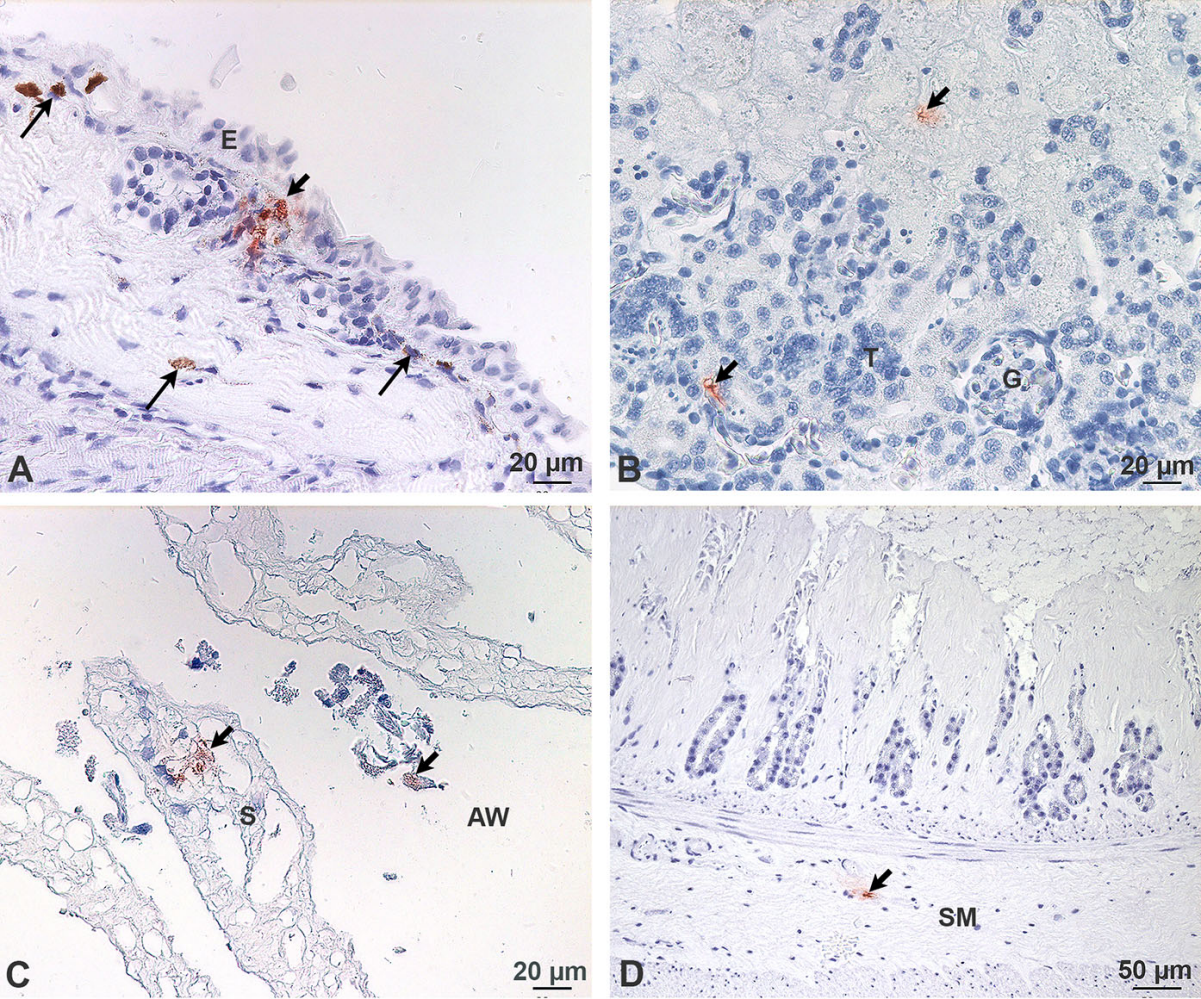
**a**–**d** Chlamydial inclusions in *Triprion spinosus* (case no. 19). A. Several chlamydial inclusions (short arrow) in the superficial dermis adjacent to the epidermis (E); dark red, granular chlamydial inclusions are distinct from dark brown deposition of variably sized pigments (long arrows). B. Chlamydial inclusions (short arrows) in interstitial cells between renal glomerula (G) and tubuli (T). C. Chlamydial inclusions (short arrows) in a pulmonary septum (S) and in a cell in the airway (AW). D. Chlamydial inclusion (short arrow) in the submucosa (SM) of the intestine. Immunohistochemistry for chlamydial LPS, counterstain hemalaun

By IHC, chlamydial inclusions were detected in tissues of *Triprion spinosus* from zoo B, but not in tissues of *Litorea caerulea* from zoo A. One of the four *Triprion spinosus* examined had chlamydial inclusions in the skin, the kidney and the intestine, and one each in the kidney and the lung only. In all of these tissues, few chlamydial inclusions were present in single cells, which were most likely macrophages. Positive cells were located in the superficial dermis of skin, in the interstitium of kidney and lung, and in the lamina propria and the submucosa of the intestine (Fig. 1). The presence of chlamydial inclusions was not associated with inflammatory infiltrates, but the frequently progressed autolysis allowed only limited evaluation of lesions. Findings are summarised in Table 4.

### Chlamydial culture and bacterial culture from clinical samples

Because of low chlamydial DNA contents resulting in cycle threshold (Ct) values above 30, only few samples were processed for chlamydial culture. All cultures gave negative results (Table 3).

Frogs from this study (Table 3; cases no. 18–23) revealed different amounts of concomitant microbiota depending on the state of decomposition. The consistent bacterial microbiota was cultivated with varying intensity on SBA and Gassner agar and identified as *Aeromonas hydrophila, Citrobacter freundii, Morganella morganii, Serratia plymuthica, Pseudomonas fluorescens, Alcaligenes faecalis, Chryseobacterium indologenes, Vagococcus fluvialis* using MALDI-TOF MS, but without any particular proofs of *Brucella* spp. or *Salmonella* spp. (data not shown).

### *Chlamydia* detection by PCR and phylogenetic analysis

Frogs from all three collections were found to be infected with *C. pneumoniae*, but definite species level identification was not attempted or did not give valid results in all cases. However, chlamydiae were detected at least once for each frog species in *intra vitam* or *post mortem* samples. Positive or questionable results were obtained from swabs taken from the skin and the mouth as well as from faeces and urine samples. Chlamydial tissue load as derived from Ct values was moderate to low (Ct 30–41) in most cases. An overview of *Chlamydia* PCR results and sample data is presented in Table 3.

**Fig. 2:**
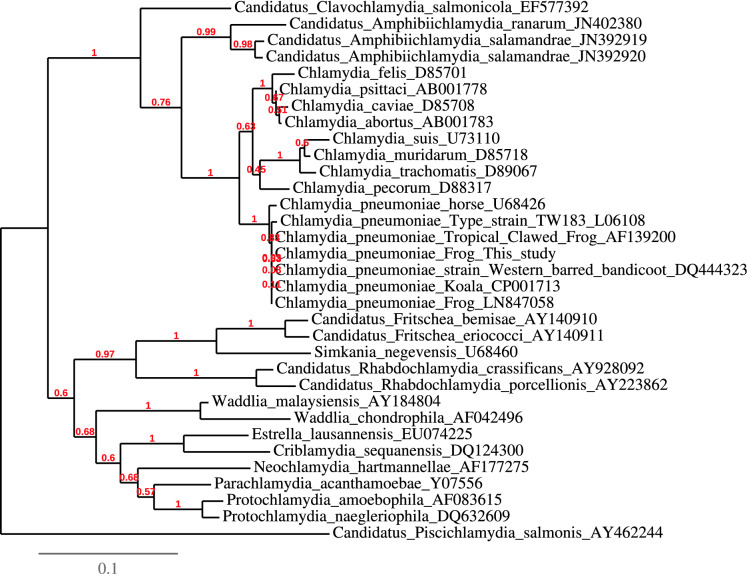
Phylogenetic tree of near full-length 16S rRNA gene sequences obtained in this study and homologues of other chlamydiae. The analysis was carried out employing PhyML software using maximum-likelihood algorithm. Bootstrap values, given at the nodes, were obtained from 100 bootstrap replicates. The scale bar represents 0.1 substitution rate

Briefly, in the private collection chlamydiae were detected only by genus specific PCR and only in tissues of *Triprion petasatus* (s. Table 3; cases no. 20–22). However, *T. spinosus* from this holding were later diagnosed with *C. pneumoniae* in zoo B. In all 13 White’s tree frogs from zoo A necropsied during 2016/2017 *C. pneumoniae* infection was diagnosed, either in faecal examinations, skin swabs or organ samples. Remarkably, all tests of swab samples or necropsied animals done in following years were negative for chlamydiae, except for one questionable skin swab. Equally, four out of five animals from the *Triprion spinosus* group quarantined in Zoo B showed positive results for chlamydiae in *post mortem* samples. In one sample from *T. spinosus* (see case 191006501; Table 3), a part of the 16S rRNA gene was amplified and sequenced (Accession No. MT487792). Sequence alignment showed 99.38–100% similarity to *C. pneumoniae* 16S rRNA gene sequences deposited in GenBank. In a phylogenetic analysis, the sequence formed one cluster with other *C. pneumoniae* sequences from different sources, while other *Chlamydia* species obtained from other amphibians (*Candidatus* Amphibiichlamydia ranarum and *Candidatus* Amphibiichlamydia salamandrae) clustered together in another clade (Fig. 2).

### Further testing

All samples were tested negative for the presence of chytrid fungus (*B*. *dendrobatidis, B. salamandrivorans*) and ranavirus by PCR (data not shown).

## Discussion

During the last 30 years, a number of studies have attempted to elucidate the role of chlamydial infections in amphibians (Table 1). The majority of cases involves the major amphibian order of frogs (Anura), but salamanders (Urodela) are also susceptible (Martel et al. 2012; Mutschmann 1998), whereas evidence in the order of caecilians (Gymnophiona) is still missing. Besides several acute mass mortality events with systemic infections, localised and chronic forms with involvement of central nervous system or lungs have been described (Berger et al. 1999; Blumer et al. 2007; Fratzke et al. 2019). Some amphibian species were found to be asymptomatically infected with chlamydiae. In the present study, the detection of chlamydiae by PCR or IHC was not correlated with specific morphological findings. In some cases, morphological alterations were attributed to other factors, for example parasitosis. However, clinical or gross pathological signs are generally not typical for chlamydiosis in amphibians (Wright 1996). Interestingly, a skin microbiome study in healthy salamanders detected chlamydiae as the fourth dominant phylotype and revealed that it makes up 24.3% of the total community (Bird et al. 2018). Potent anti-chlamydial dermaseptins have also been found in salamander skin. It was shown that they were highly effective against *C. trachomatis*, reducing the numbers of inclusion-forming units and blocking novel infections in vitro (Bergaoui et al. 2013). Although infections do occur in the wild (Berger et al. 1999; Blumer et al. 2007; Martel et al. 2013), this could suggest a finely tuned balance mechanism under natural conditions. On the other hand and in the light of the worldwide amphibian decline, chlamydiae have been discussed to possess the potential to cause considerable impact on amphibian health (Martel et al. 2012). It remains to be determined, if and to which extent environmental or maintenance circumstances and cofactors influence the clinical outcome of amphibian chlamydiosis and asymptomatic chlamydial infections and whether such factors contribute to the microorganism’s role as an obligate or facultative pathogen.

All previous studies have found chlamydiae only following *post mortem* examinations, but—like e.g. in reptiles (Rüegg et al. 2015; Taylor-Brown et al. 2015) and as obtained from skin, mouth, faecal and urine samples from the current study—*intra vitam* diagnosis is also possible in amphibians (Table 3). Unfortunately, it was not possible to properly identify the involved chlamydiae at species level in all cases because tissue material had not been stored in retrospective cases. Likewise, bacterial load in tissue samples might have been too low in other cases, in which amplification or isolation in cell cultures were unsuccessful. It is tempting to speculate, that infection series within the same facility were caused by the same chlamydial species. *C. pneumoniae* has in the past been by far the predominant chlamydial species in amphibians and reptiles according to literature (Staub et al. 2018). This pathogen is usually found in humans and most adults become infected with *C. pneumoniae* at least once during their lifetimes (Aldous et al. 1992). Other susceptible host species are ruminants, pigs, horses, koalas and reptiles (Berger et al. 1999; Blumer et al. 2007; Bodetti et al. 2002). Possibly, the spectrum of chlamydial species in amphibians is like in reptiles more diverse than known so far. There are other phylogenetically related, recently described reptilian taxa like *C. serpentis* and *Candidatus* C. testudinis, C. corallus and C. sanzinia which represent uncultivable or difficult to culture chlamydia (Laroucau et al. 2020; Staub et al. 2018; Taylor-Brown et al. 2016, 2017). In this context, the present report elucidates the importance of including routine testing on *Chlamydia* in amphibians. Culture-independent methods like metagenomics methods will support detection of uncultivable and hitherto unknown chlamydial species (Taylor-Brown et al. 2017).

In the present study, clinical signs like lethargy, anorexia, bloating as well as faded and sloughing skin were also observed (Table 2), but a very uncommon behaviour was regurgitation within 2 days after feeding that has previously only once been described for reptiles (Bodetti et al. 2002). Frogs in this study often lost their vitality, stopped calling and breeding and showed a state of chronic wasting until death after months. A poor body condition at necropsy accompanied by absent fat bodies and empty intestinal tracts was typical and has been described before (Blumer et al. 2007). In order to improve the health status several antimicrobial treatment regimens have been carried out in some of the here mentioned amphibian species groups. To avoid stress of daily handling of each single frog, bathing treatments with different medications were chosen. This apparently convenient application of antimicrobials has some pitfalls because pharmacokinetics and pharmacodynamics are largely unknown for the different species and most frogs tend to leave the body of water treatment. Therefore, a raining system used for breeding was utilised with prolonged contact times to ensure adequate absorption of the drugs through the skin and bladder wall. However, all the attempted treatments were unsuccessful because clinical signs did not improve and chlamydiae were still detected. Description of therapy regimes has rarely been included in papers published on amphibian chlamydiosis. Staub and colleagues found their reptilian isolates of *C. serpentis* and *C. poikilothermis* being temperature-sensitive and in vitro susceptible to tetracycline and moxifloxacin, but intermediate to resistant to azithromycin (Staub et al. 2018). Likewise, treatment was unsuccessful or associated with long-term neurologic deficits so that depopulation of tanks housing infected animals was used to control the disease (Fratzke et al. 2019). Rüegg et al. (2015) report treatment of seven snakes with chlamydiosis for up to 35 days with a daily intramuscular injection of marbofloxacin and could show on the one hand successful treatment confirmed by negative PCR, but on the other hand one loss and one therapy-resistant snake suggesting general need for implementation of safer and more efficient treatment protocols.

Previous studies have used a plethora of methods for the diagnosis of amphibian chlamydial infections (Table 2). Chlamydial culture was not successful in our case series, which might have been caused by low multiplicity of infection, possibly due to chronic state or by use of inadequate cell lines and culture conditions. Ct values above 32 are considered as an indicator for insufficient culture results (CS; personal experience). A low number of infected cells could be confirmed by IHC in some animals (Fig. 1).

Chlamydiae are prone to enter a phase of intracellular persistence when exposed to unfavourable conditions leading to remaining in a viable but non-cultivable, one could also refer to it as a “dormant” state. This can explain unsuccessful antimicrobial treatment and in vitro cultivation. Hence, animals are reported to stay persistently infected for longer time periods and remain in an asymptomatic carrier status resulting in persistent, asymptomatic or minimally symptomatic, chronic infections. Those infections have the character of wasting diseases in the long term due to tissue damage in the pathogenesis of chlamydial disease (Borel et al. 2018; Panzetta et al. 2018). Eventually, clinically inapparent low-level chlamydial infections have been linked even with symbiosis (Horn 2008; Martel et al. 2013). Due to these unique features of “arrested” chlamydial replication and multiplication decreased detection sensitivity is to be expected.

Interestingly, further analysis particularly of the *ompA* and other genes of chlamydiae originating from poikilothermic animals revealed high similarity or even identical sequences to chlamydiae associated with respiratory and cardiovascular disease in humans (Bodetti et al. 2002; Cochrane et al. 2005). Meanwhile, with more in-depth whole genome sequencing (WGS)-based techniques becoming available, a growing body of evidence shows that human *C. pneumoniae* strains have once been zoonotically acquired and may have evolved from poikilothermic animals resulting in human-adapted chlamydiae becoming independent of the animal reservoir (Mitchell et al. 2010; Myers et al. 2009). Based on a multiple gene analysis from 30 *C. pneumoniae* strains from humans and animals (including amphibians) it was concluded that they belong to five genotypes (A–E), from which genotypes A, B, and C are among others associated with frogs (Mitchell et al. 2010). The authors hypothesise that a basal clade of amphibian isolates (cf. (Bodetti et al. 2002; Hotzel et al. 2001; Reed et al. 2000); genotype C) evolved to initially infect reptilian hosts and now represents the dominant clonal lineage of human infections (Mitchell et al. 2010). Interestingly, a second lineage has been identified with amphibian isolates (cf. Berger et al. 1999; Blumer et al. 2007; genotype A) as a starting point that evolved into two sub-lineages, one infecting Australian marsupials while the other branch has once more crossed the animal-human barrier to infect Australian Aboriginals (Mitchell et al. 2010). Similar results could be obtained by WGS analysis (Roulis et al. 2015).

## Conclusion

The aim of the present study is to raise awareness about chlamydial infections in amphibians, especially frogs, and to suggest a targeted analysis in routine diagnostics. Among the *Chlamydia* spp. especially *C. pneumoniae*, but also so far unclassified species must be considered in the differential diagnosis of infectious amphibian diseases. Hence, we observed in our study an association between clinical signs of infertility and chronic wasting and pathological findings with infections caused by chlamydial organisms in frogs. Treatment should be only initiated, if chlamydia are confirmed as causative pathogens, since current treatment options have a poor prognosis due to possible persistence of this pathogen. Although *C. pneumoniae* is considered a ‘monomorphic pathogen’ with high intraspecific genetic similarity and human-to-human infections are frequently observed, *C. pneumoniae* isolates from amphibians do not seem to have a pronounced zoonotic potential.

## Data Availability

All data has been made fully available to the public.
